# Anti-inflammatory properties of commonly used psychiatric drugs

**DOI:** 10.3389/fnins.2022.1039379

**Published:** 2023-01-10

**Authors:** Shrujna Patel, Brooke A. Keating, Russell C. Dale

**Affiliations:** ^1^Faculty of Medicine and Health, Kids Neuroscience Centre, The Children's Hospital at Westmead, University of Sydney, Westmead, NSW, Australia; ^2^Faculty of Medicine and Health, Clinical School, The Children's Hospital at Westmead, University of Sydney, Westmead, NSW, Australia; ^3^Faculty of Medicine and Health, Sydney Medical School, University of Sydney, Camperdown, NSW, Australia

**Keywords:** inflammation, anti-depressants, anti-psychotics, mood stabilizers, mental health

## Abstract

Mental health and neurodevelopmental disorders are extremely common across the lifespan and are characterized by a complicated range of symptoms that affect wellbeing. There are relatively few drugs available that target disease mechanisms for any of these disorders. Instead, therapeutics are focused on symptoms and syndromes, largely driven by neurotransmitter hypotheses, such as serotonin or dopamine hypotheses of depression. Emerging evidence suggests that maternal inflammation during pregnancy plays a key role in neurodevelopmental disorders, and inflammation can influence mental health expression across the lifespan. It is now recognized that commonly used psychiatric drugs (anti-depressants, anti-psychotics, and mood stabilizers) have anti-inflammatory properties. In this review, we bring together the human evidence regarding the anti-inflammatory mechanisms for these main classes of psychiatric drugs across a broad range of mental health disorders. All three classes of drugs showed evidence of decreasing levels of pro-inflammatory cytokines, particularly IL-6 and TNF-α, while increasing the levels of the anti-inflammatory cytokine, IL-10. Some studies also showed evidence of reduced inflammatory signaling *via* nuclear factor- (NF-)κB and signal transducer and activator of transcription (STAT) pathways. As researchers, clinicians, and patients become increasingly aware of the role of inflammation in brain health, it is reassuring that these psychiatric drugs may also abrogate this inflammation, in addition to their effects on neurotransmission. Further studies are required to determine whether inflammation is a driver of disease pathogenesis, and therefore should be a therapeutic target in future clinical trials.

## 1. Introduction

Mental health disorders are extremely common across the lifespan, affecting many people from childhood through to old age. Neurodevelopmental disorders, such as autism spectrum disorder (ASD), attention deficit hyperactivity disorder (ADHD), Tourette syndrome (TS), and obsessive-compulsive disorder (OCD) affect 10% of all children and are a leading cause of disability globally (Global Research on Developmental Disabilities Collaborators, 2018). Neurodevelopmental disorders often co-occur with mental health disorders, namely major depressive disorder (MDD) and anxiety, resulting in a complicated range of symptoms that can affect wellbeing (Salazar et al., 2015; Hansen et al., 2018). Adolescents are particularly vulnerable to mental health disorders, often associated with environmental stress, trauma, and substance abuse (Shanahan et al., 2008; Merikangas et al., 2010; Schulte and Hser, 2014), leading to poorer life outcomes in adulthood (Gibb et al., 2010). Major depression, bipolar disease, and schizophrenia are common in adulthood, and are considered some of the costliest disorders to humanity. People with dementia are also at high risk of developing co-existing psychiatric and behavioral disturbances, requiring supportive care (Kales et al., 2005, 2015). Collectively, neurodevelopmental and mental health disorders reduce quality of life for a significant proportion of the population and place an enormous economic burden on society (Greenberg et al., 2015; Leigh and Du, 2015).

Although genetic contributions are manifest in mental health disorders (Cross-Disorder Group of the Psychiatric Genomics Consortium, 2019), highly penetrant genetic variations are uncommon. Instead, a combination of genetic vulnerability and environmental factors is the more commonly accepted model of disease (Tsuang et al., 2004; Caspi and Moffitt, 2006). Despite intensive research efforts, there are relatively few drugs available that target disease mechanisms for any of these disorders (Wong et al., 2010). Instead, therapeutics are driven by symptoms and syndromes, often characterized by Diagnostic and Statistical Manual of Mental Disorders (DSM) or International Classification of Diseases (ICD) criteria.

Several serendipitous findings of the mid-20th century gave rise to the field of modern neuropharmacology and revolutionized the way in which specific symptoms of an array of disorders were managed. Following observations of the CNS-modulating effects of chlorpromazine as an anesthetic, it was supplied to a small group of psychiatrists who trialed the drug in schizophrenic and manic patients (Shen, 1999). Symptomatic relief and functional improvement in patients, paired with a global decrease of psychiatric inpatient admissions, rapidly confirmed chlorpromazine as a drug of profound clinical significance. Following the success of chlorpromazine, molecular modification of compounds with similar chemical structures gained momentum. Imipramine, developed as a weak anti-histamine with mild anti-cholinergic effects, proved ineffective in schizophrenia, however early researchers recognized the potential mood modifying effects of the drug and it was released for clinical use within a year of its first publication (Pereira and Hiroaki-Sato, 2018). Parallel to these studies, the clinical effects of iproniazid were also being investigated, with the observation this drug greatly stimulated the CNS initially being listed as a side effect, before the potential of this stimulation was realized in the context of depression (Pereira and Hiroaki-Sato, 2018).

The studies of the early 1950s and 1960s laid the groundwork for the development of many distinct classes of drugs in neuropsychology, as well as neurotransmitter-specific hypotheses of pathogenesis related to serotonin, dopamine, glutamate, noradrenaline, GABA (γ-aminobutyric acid), and acetylcholine (Wong et al., 2010). For example, the serotonin hypothesis in emotional disorders, such as anxiety, depression, and OCD, has supported the development of serotonergic drugs in these syndromes, such as selective serotonin reuptake inhibitors (SSRIs), which are now the first line medication of choice for emotional disorders (Vaswani et al., 2003). Despite this, the evidence for serotonergic dysfunction in emotional disorders lacks definitive acceptance (Nordquist and Oreland, 2010; Gardner and Boles, 2011). Likewise, in psychosis, dopaminergic and glutamate hypotheses are supported by multiple lines of evidence, but the origins of these neurotransmitter dysfunctions are unclear (Stahl, 2018). The main classes of psychiatric drugs, namely anti-depressants, anti-psychotics, and mood stabilizers are used in both children and adults to treat a wide range of neurodevelopmental and mental health problems. It is now widely accepted that inflammation plays a role in many of these health problems, and many studies have documented the therapeutic effects of anti-inflammatory drugs in psychiatric disorders (Miller and Raison, 2015; Müller, 2019; Fitton et al., 2022).

## 2. The role of inflammation across the lifespan

In parallel to this literature, there is emerging evidence to support the role of inflammation in neurodevelopmental and mental health disorders. The immune system plays a critical role in both health and disease across the lifespan (Bilbo and Schwarz, 2009; Furman et al., 2019). Immune dysregulation has been reported in all major syndromes, including ASD, ADHD, OCD, TS/tics, MDD, anxiety, schizophrenia, and psychosis (Ashwood et al., 2011; Mitchell and Goldstein, 2014; Young et al., 2014; Masi et al., 2015; Radhakrishnan et al., 2017). Many environmental factors associated with mental health disorders are known to be pro-inflammatory, such as stress (Rohleder, 2019) and chronic disease (Pawelec et al., 2014). Furthermore, the maternal immune activation hypothesis proposes a link between maternal inflammation during pregnancy and increased risk of neurodevelopmental and neuropsychiatric disorders in offspring (Han et al., 2021a). Evidence shows that pro-inflammatory disorders affecting the mother during pregnancy, such as autoimmunity, infection, asthma, obesity, and gestational diabetes are associated with increased risk of ASD, ADHD, and TS in offspring (Han et al., 2021b). Ongoing pro-inflammatory factors such as stress, obesity, diet, exercise, smoking, pollution, and chronic disease continue to play a role in adulthood and may have causal or exacerbating influences (Furman et al., 2019). Therefore, inflammation resulting from environmental risk factors (e.g., stress, trauma, chronic disease) may play a role in expression of disease throughout the lifespan, from pre-conception, pregnancy, childhood, and into adulthood.

The main psychiatric drugs commonly used in neurodevelopmental and mental health disorders (in both children and adults), such as anti-depressants, anti-psychotics, and mood stabilizers, were designed for their neurotransmitter effects. However, it is now recognized that these drugs have multiple mechanisms of action, including anti-inflammatory effects. Animal models have been developed to study the effects of inflammation on behavior, such as the lipopolysaccharide (LPS) challenge or the interleukin (IL)-1β-induced model of depression in mice. These models have shown that many psychiatric drugs have anti-inflammatory characteristics (reviewed thoroughly by Song and Wang, 2011 and Ma et al., 2017). However, debate continues as to the translational validity of these animal studies to human disease (Barroca et al., 2022).

Here, we have reviewed the human evidence regarding the anti-inflammatory properties of commonly used psychiatric drugs. We present this literature in a narrative review, divided by major drug subgroups, starting with SSRIs, then anti-psychotics and mood stabilizers, followed by other remaining psychiatric drugs. There have been several systematic reviews or meta-analyses (discussed further in subsequent sections) that focus on classes of drugs in certain disorders with specific outcome measures; for example, the effect of anti-psychotics on peripheral cytokine levels in schizophrenia (Tourjman et al., 2013). Therefore, we have not conducted a systematic review. Rather, aim to address a gap in the literature by drawing together the human evidence of anti-inflammatory mechanisms for the main classes of psychiatric drugs across a broad range of mental health disorders.

## 3. Overview of the investigation of anti-inflammatory mechanisms of psychiatric drugs

Tables 1–6 capture some of the existing investigation into the effects of psychiatric drugs on immune function in humans. The methods that have been used to examine these anti-inflammatory properties are disparate, and we separate them into two main types of methodology: *in vivo* peripheral immune studies and *ex vivo/in vitro* cell culture studies. The *in vivo* peripheral immune studies focus primarily on measuring peripheral cytokine levels in the blood as markers of inflammation. These studies compare cytokine levels between patients with psychiatric disorders and controls at baseline, as well as between patient cohorts before and after treatment. The pro-inflammatory cytokines most commonly measured to investigate these effects are IL-1β, IL-6, interferon (IFN) species, and tumor necrosis factor (TNF)-α, while IL-4 and IL-10 are the most commonly measured anti-inflammatory cytokines. The *ex vivo/in vitro* cell culture studies involve isolating and culturing immune cells from patients with psychiatric disease or healthy controls and examining the effects of drugs through functional analyses of cell signaling pathways, or cytokine production.

**Table 1:** Anti-inflammatory effects of SSRIs and/or SNRIs in human studies.

**Drug name**	**Population**	**Method**	**Results: Difference in patients (vs. controls) at baseline, or patients pre- and post-treatment**	**References**
Fluvoxamine, sertraline, paroxetine, and milnacipran	MDD (*n* = 51, 28 females, mean age 40 years) Healthy controls (*n* = 30, sex- and age- matched)	ELISA plasma for BDNF, IL-6, and TNF-α. Anti-depressants grouped together for statistical analyses.^*^	At baseline: ↑ IL-6 (highest in refractory group) and ↓ BDNF in patients. On treatment: ↑ IL-6 levels (but not in refractory group) and ↓ BDNF. TNF-α unchanged both treatment groups.	Yoshimura et al., 2009
Sertraline	First-episode MDD (*n* = 23, 11 females, mean age 34.7 years) Healthy controls (*n* = 25, 12 females, mean age 34.3 years)	ELISA serum for IL-2, IL-4, IL-12, TGF-β1, TNF-α, and MCP-1.	At baseline: ↑ IL-2, IL-12, TNF-α, and MCP-1. ↓ IL-4 and TGF-β. On treatment: ↑ IL-12, and ↓ IL-4 and TGF-β1.	Sutcigil et al., 2007
Sertraline	MDD (*n* = 20, 13 females, mean age 37 years) Healthy controls (*n* = 20, 12 females, mean age 36.4 years)	ELISA serum for IL-6 and IL-10. Thin layer liquid chromatography, gas chromatography/mass spectrometry to measure F2-IsoPs (marker of oxidative stress).	At baseline: No difference in cytokines. In MDD, F2-IsoP concentrations significantly positively correlated with IL-6 levels, and negatively with IL-10. On treatment: No correlation remaining between F2-IsoPs and IL-6 or IL-10, suggesting some resolution of oxidative stress/inflammation post treatment.	Rawdin et al., 2013
Sertraline, escitalopram	First-episode GAD (*n* = 42, 18–60 years)	ELISA serum for IL-1β, IL-6, IL-8, IL-12p70, and IFN-γ. Turbidimetry-based assay to measure CRP.	On treatment: ↓ IL-1β, IL-6, IL-8, IL-12p70, IFN-γ, and CRP.	Hou et al., 2019
Escitalopram	MDD patients (*n* = 20, 16 females, mean age 37.1 years) Healthy controls (*n* = 27, 20 females, mean age 38.4 years)	Liquid or gas chromatography for serum TRP, KYN, KYNA, 3HK, and QUINA. Biochip array and ELISA validation for plasma IL-1α, IL-1β, IL-4, IL-6, IL-8, IL-10, MCP-1, CRP, and TNF-α.	At baseline: ↓ CRP, TNF-α, IL-6, and MCP-1. On treatment: No significant differences in cytokines. ↓ 3HK (12 weeks Tx); ↓ QUINA (8 weeks Tx); ↑ KYN:QUINA ratio (8 weeks Tx); ↓ QUINA:TRP ratio (8 weeks Tx); ↓ QUINA:3HK ratio (8 weeks Tx).	Halaris et al., 2015
Escitalopram	MDD patients (*n* = 100, 57 females, mean age 32.1 years) Healthy controls (*n* = 45, 26 females, mean age 32.9 years)	Enzyme-labeled chemiluminescence for serum sIL-2R, IL-8, and TNF-α.	At baseline: ↓ IL-8 and ↑ sIL-2R. Drug responders had ↓ TNF-α. On treatment: ↓ sIL-2R in non-responders at 4 weeks on drug, lost by 12 weeks. No other significant differences.	Eller et al., 2008
Escitalopram, nortriptyline	Moderate-severe MDD (*n* = 90, 64 females, mean age 38 years)	Multiplex ELISA for serum levels of 27 cytokines.	On treatment: ↓ IL-2, IL-4, IL-7, IL-8, IL-9, IL-13, IL-17, eotaxin-1, G-CSF, IP-10, MCP-1, PDGF-bb, MIP-1b, RANTES, and TNF-α.	Kofod et al., 2022
Duloxetine	MDD patients (*n* = 16, 12 females, mean age 51.1 years) Healthy controls (*n* = 16, nine females, mean age 44 years)	ELISA serum for IL-6.	On treatment: No overall difference in IL-6 levels. Responders ↑ IL-6.	Fornaro et al., 2011
Duloxetine	Fibromyalgia patients (*n* = 128, all female, mean age 41.6 years)	ELISA for serum IL-6, IL-8, and TNF-α.	On treatment: ↓ IL-6 and IL-8. No change in TNF-α levels.	Zabihiyeganeh et al., 2019
Escitalopram, venlafaxine, citalopram, mirtazapine, sertraline^**^	MDD patients (*n* = 50, 38 females, mean age 40 years) Healthy controls (*n* = 34, 19 females, mean age 38.3 years)	Cytokine assay for plasma IL-1β, IL-1, IL-1Ra, IL-2, IL-5, IL-6, IL-7, IL-8, IL-10, IL-15, G-CSF, MIP-1-α, TNF-α, and IFN-γ.	At baseline: ↑ IL-1β, IL-1Ra, IL-5, IL-6, IL-7, IL-8, IL-10, G-CSF, and IFN-γ. On treatment: ↓ IL-1Ra, IL-6, IL-7, IL-8, IL-10, G-CSF, and IFN-γ.	Dahl et al., 2014

* Groups analyzed were SSRI/SNRI responsive, SSRI/SNRI refractory, or healthy control.

** Some patients also received additional lamotrigine.

MDD, major depressive disorder; ELISA, enzyme-linked immunosorbent assay; BDNF, brain-derived neurotrophic factor; IL, interleukin; TNF, tumor necrosis factor; TGF, transforming growth factor; MCP, monocyte chemoattractant protein; GAD, generalized anxiety disorder; IFN, interferon; CRP, C-reactive protein; TRP, tryptophan; KYN, kynurenine; KYNA, kynurenic acid; 3HK, 3-hydroxy-kynurenine; QUINA, quinolinic acid; Tx, treatment; sIL-2R, soluble interleukin-2 receptor; G-CSF, granulocyte-colony stimulating factor; IP, induced protein; PDGF-bb, platelet-derived growth factor with two B subunits; MIP, macrophage inflammatory protein; RANTES, regulated on activation; normal T cell expressed and secreted; IL-1Ra, IL-1 receptor antagonist.

### 3.1. Selective serotonin reuptake inhibitors

SSRIs are common first-line therapeutics for a variety of emotional and psychiatric conditions, such as MDD, OCD, and anxiety disorders. More recently, another class of drug with additional actions on the noradrenaline system, termed SNRIs (serotonin-norepinephrine reuptake inhibitors), have been developed. SNRIs are now also common first-line anti-depressant therapeutics with effects in anxiety, depression, and OCD, but there is also some evidence of utility in pain syndromes and fibromyalgia (Zabihiyeganeh et al., 2019). When first developed, SSRIs revolutionized treatment of mood disorders by targeting monoaminergic systems, but there is increasing evidence that SSRIs and SNRIs can also modulate inflammation and immune activation, as summarized in Table 1. Notably, these studies have been performed solely in adults (rather than children), and largely in the context of depression, with a small number in anxiety and pain disorders. Most of these studies compared serum or plasma cytokines (single cytokines or panels of cytokines) at baseline in patients with controls, and then compared cytokines on treatment compared to baseline.

The main theme is that SSRIs/SNRIs can reduce expression of pro-inflammatory cytokines, particularly IL-6 and TNF-α (Table 1). As well as cytokines, metabolites in the kynurenine pathway can provide valuable insight into cellular function and inflammation: briefly, kynurenine is increased in inflammation, kynurenic acid (KYNA) is an anti-inflammatory and neuroprotective metabolite, and conversely quinolinic acid (QUINA) is a pro-inflammatory and neurotoxic metabolite produced within this pathway. An elegant study from Halaris et al. (2015) in patients with MDD showed treatment with the SSRI escitalopram ameliorated neurotoxicity by increasing the ratio of neuroprotective KYNA to QUINA and decreasing the ratio of QUINA to tryptophan. A study from Borsini et al. (2017) builds on these findings (Table 2) and showed incubation with the SSRI sertraline *in vitro* also reduces levels of QUINA and other enzymes in the kynurenine pathway.

**Table 2:** Anti-inflammatory effects of SSRIs and/or SNRIs using *in vitro* or *ex vivo* human cells in culture or stimulation assays.

**Drug name**	**Cell type (cell line or human source)**	**Immunological or cellular method**	**Results *in vitro*/*ex vivo*: Difference following application of drug**	**References**
Fluoxetine, paroxetine, bupropion	A375 or HEPG2 cell lines	Differential gene expression (transcriptomic analysis) signatures produced by each drug.	SSRI altered gene expression, similar to *IL6ST* and *NFKB1* gene knockdown cells.	Creeden et al., 2021
Fluoxetine	Placental explant cultures treated with 0–200 μM drug in presence or absence of heat-killed *E. coli*	Cytokine assay for IL-1β, TNF-α, HO-1, IL-10, IL-6, and BDNF.	No change in IL-1β, TNF-α, and IL-10. ↓ BDNF at concentrations 12–200 μM.	Clementelli et al., 2021
Fluoxetine	BV-2 cells subjected to oxygen glucose deprivation/reoxygenation (model of ischemic brain injury)	ELISA for TNF-α, IL-1β, and IL-6. RT-PCR to assess IκB-α RNA expression. Western blot for NF-κB-signaling proteins. DARTS assay for detecting interaction between drug and IκB-α.	Dose-dependent ↓ TNF-α, IL-1β, and IL-6 in culture supernatant and ↓ phosphorylation of NF-κB subunits. Dose-dependent ↑ in IκB-α protein, negative regulator of NF-κβ signaling.	Tian et al., 2019
Fluvoxamine	LPS-stimulated HUVECs and Human U937 macrophages	RT-PCR for ICAM-1, VCAM-1, COX2, iNOS.	HUVECS: ↓ VCAM-1, ICAM-1, iNOS, and COX2 with fluvoxamine. U937 macrophages: ↓ iNOS and COX2 with fluvoxamine.	Rafiee et al., 2016
Fluvoxamine, clomipramine, nortriptyline, fluoxetine	Healthy volunteers—PMNs	Boyden chamber to measure chemotaxis.	Clomipramine and nortriptyline dose -dependent ↓ PMN chemotaxis. Fluvoxamine and fluoxetine no effect on PMN chemotaxis.	Sacerdote et al., 1994
Clomipramine, desipramine, fluoxetine, reboxetine, trimipramine	Whole blood cultures stimulated with LPS (to stimulate monocyte-derived cytokines) or Con A (to stimulate T cell cytokines) Healthy volunteers (*n* = 8, five females, mean age 22.6 years)	ELISA for supernatant IL-1β, IL-12, TNF-α, IFN-γ, and IL-10. Liquid scintillation counter used to measure lymphocyte proliferation.	All drugs ↑ stimulation-induced IFN-γ. ↓ IL-10 with desipramine. All drugs reduced T cell proliferation.	Diamond et al., 2006
Clomipramine	Whole blood cultures stimulated with LPS and PHA Healthy volunteers (*n* = 9, four females, mean age 33.1 years)	ELISA for supernatant IFN-γ and IL-10.	Treatment ↓ IFN-γ and ↑ IL-10.	Maes et al., 1999
Clomipramine	MDD patients (*n* = 15, 11 females, mean age 49 years) Healthy controls (*n* = 28, 21 females, mean age 45 years)	Whole blood cultures incubated with clomipramine and stimulated with LPS for glucocorticoid assay. Immunoassay for plasma cortisol and IL-6.	On treatment: ↑ plasma IL-6 levels. ↓ IL-6 levels after *in vitro* drug treatment both in the absence and presence of LPS stimulation.	Carvalho et al., 2008
Sertraline, venlafaxine	Multipotent human hippocampal progenitor cell line HPC0A07/03C Cells cultured with IL-1β either alone or with sertraline or venlafaxine	Immunocytochemistry for differentiation and maturation assay. qPCR for IDO, KMO, KYNU, and ACMSD. Liquid chromatography for TRP, KYN, anthranilic acid, picolinic acid, and QUINA.	Both drugs protect against IL-1β-induced reduction in neurogenesis. Sertraline ↓ QUINA and ↓ KMO mRNA.	Borsini et al., 2017

IL, interleukin; TNF, tumor necrosis factor; HO, heme oxygenase; BDNF, brain-derived neurotrophic factor; ELISA, enzyme-linked immunosorbent assay; RT-PCR, reverse transcription polymerase chain reaction; RNA, ribonucleic acid; NF-κβ, nuclear factor κβ; DARTS, drug affinity responsive target stability; LPS, lipopolysaccharide; HUVECS, human umbilical vein endothelial cells; ICAM, intercellular adhesion molecule; VCAM, vascular cell adhesion molecule; COX, cyclo-oxygenase; iNOS, inducible nitric oxide synthase; PMN, polymorphonuclear cells; IFN, interferon; PHA, phytohemagglutinin; qPCR, quantitative polymerase chain reaction; IDO, indoleamine 2; 3-dioxygenase; KMO, kynurenine 3-monooxygenase, KYNU, kynureninase; ACMSD, aminocarboxymuconate semialdehyde decarboxylase; TRP, tryptophan; KYN, kynurenine; QUINA, quinolinic acid.

**Figure 1:**
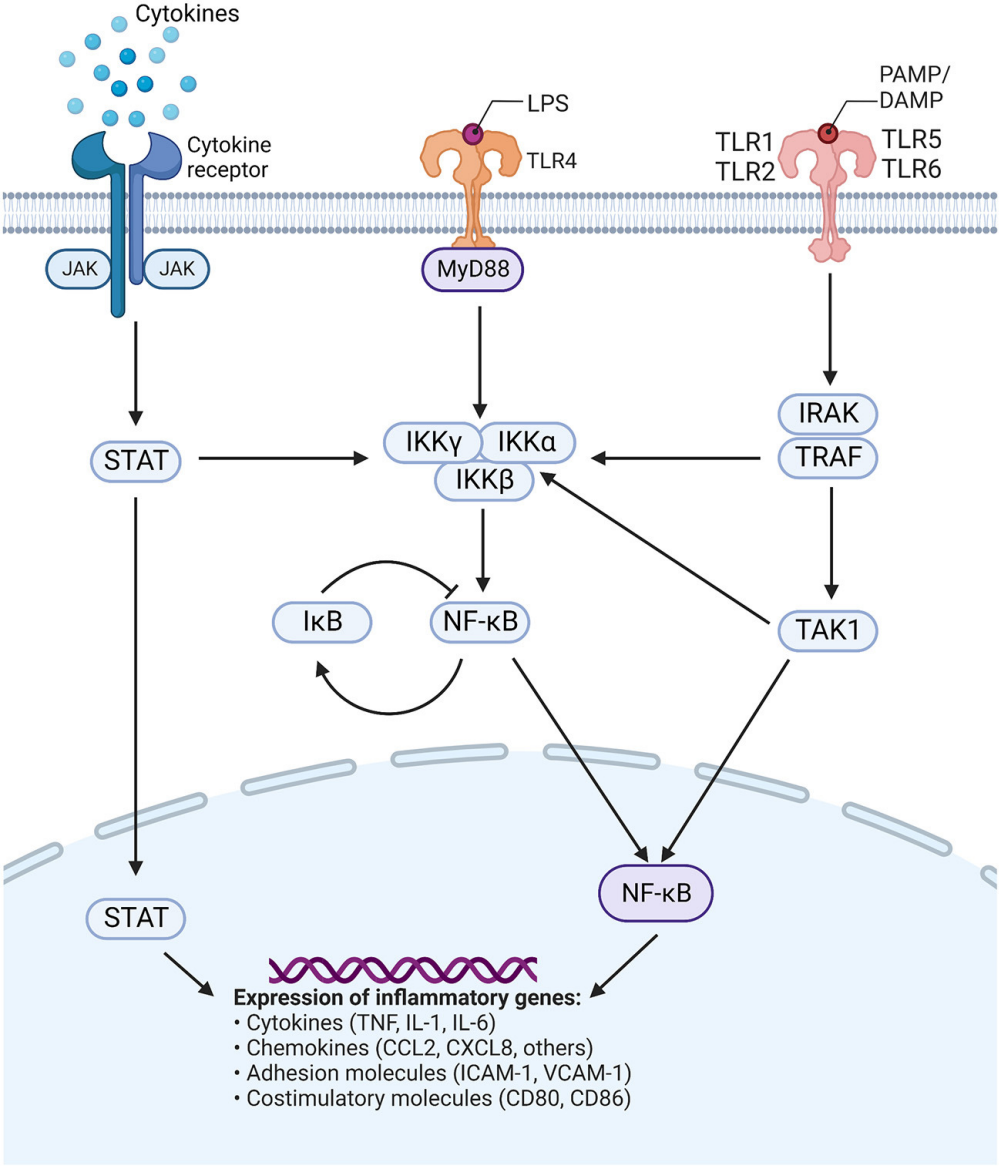
Common signaling pathways. TLRs are activated by various stimuli, commonly recognizing viral and bacterial products, or host-derived endogenous compounds (i.e., DAMPs or PAMPs, respectively). Triggering of TLRs can activate multiple pathways which produce complexes that activate the IKK structure (comprising catalytic IKKα and IKKβ, and regulatory IKKγ subunits). NF-κB is produced and either translocates to the nucleus or is negatively regulated by phosphorylation of IκB. In the nucleus, NF-κB influences the expression of many inflammatory genes, in turn affecting production of cytokines, chemokines, adhesion molecules, and costimulatory molecules. Activation of the JAK/STAT pathway *via* cytokine receptors also contributes to NF-κB signaling and alterations in the expression of inflammatory genes. JAK, Janus kinase; STAT, signal transducer and activator of transcription; TLR, toll-like receptor; DAMP, damage-associated molecular patterns; PAMP, pathogen-associated molecular pattern; MyD88, myeloid differentiation primary response 88; IKK, inhibitor of κB kinase; IRAK, interleukin receptor-associated kinase; TRAF, tumor necrosis factor receptor-associated factor; IκB, inhibitor of nuclear factor κB; NF-κB, nuclear factor κB; TNF, tumor necrosis factor; IL, interleukin; CCL, CC motif chemokine ligand;; CXCL, CXC motif chemokine ligand; ICAM, intercellular adhesion molecule; VCAM, vascular cell adhesion molecule; CD, cluster of differentiation. Figure adapted from “TLR Signaling Pathway”, by BioRender.com (2015). Retrieved from: https://app.biorender.com/biorender-templates.

Similarly, many *in vitro* studies have investigated inflammatory cytokine expression following SSRI/SNRI treatment (Table 2). In various *in vitro* models of inflammation, SSRIs have been shown to reduce inflammatory cytokines such as IL-6, TNF-α, IFN-γ, and IL-1, while often increasing levels of the anti-inflammatory IL-10. A study by Creeden et al. (2021) also demonstrated the ability of SSRIs to disrupt nuclear factor- (NF-)κB signaling, in turn reducing transcriptional activation of IL-6 and ameliorating the “cytokine storm” often seen with acute inflammation (see Figure 1 for schematic of common signaling pathways). Likewise, in an *in vitro* model of ischemic brain injury it was shown that, while decreasing inflammatory cytokine levels, the SSRI fluoxetine also decreased levels of NF-κB subunits through dose-dependent upregulation of the protein IκB, a negative regulator of the NF-κB signaling pathway (Tian et al., 2019) (Figure 1). SSRIs are also able to inhibit expression of inflammatory genes related to adhesion molecules ICAM-1 and VCAM-1 (intracellular cell adhesion molecule-1 and vascular cell adhesion molecule-1, respectively), which are usually upregulated on vascular endothelium and leukocytes during inflammatory events. Furthermore, SSRIs/SNRIs also reduce expression of inflammatory mediators COX2 and iNOS (cyclo-oxygenase 2 and inducible nitric oxide synthase, respectively) (Rafiee et al., 2016). Additionally, a small number of studies have shown SSRIs can reduce polymorphonuclear chemotaxis (Sacerdote et al., 1994) and reduce T cell proliferation (Diamond et al., 2006). Systematic reviews and meta-analyses have also confirmed the modulatory effects of SSRIs and SNRIs in inflammatory contexts. One meta-analysis concluded that peripheral levels of IL-6, TNF-α, and IL-10 were decreased following SSRI therapy (Köhler et al., 2017), with another similarly showing reductions in plasma TNF-α but not IL-6 (Almeida et al., 2020); this second meta-analysis used stringent inclusion criteria, and the authors acknowledge that very few studies met their inclusion criteria which may have contributed to this conflicting result.

### 3.2. Anti-psychotics

Anti-psychotics, such as risperidone, olanzapine, and aripiprazole, are primarily used to treat schizophrenia and psychosis (Gardner, 2005); however, there is first-level evidence of their effect in treating behavioral disturbance in people with ASD (Aman et al., 2008; Posey et al., 2008). Anti-psychotics have many mechanisms of action, although their primary proposed action is to alter dopaminergic neurotransmission in the brain (Horacek et al., 2006). Clozapine, a major pharmacological agent in managing treatment-resistant schizophrenia, is distinct in its mechanism of action, antagonizing dopamine receptors while also binding with high affinity to several serotonin receptors, histamine receptors, adrenergic receptors, and muscarinic receptors (Gammon et al., 2021). Studies investigating the effects of anti-psychotics on inflammation have largely been conducted in adults with schizophrenia or psychosis (Table 3). Most of these studies measured the effect of anti-psychotic treatment on serum cytokine/chemokine levels using enzyme-linked immunosorbent assays (ELISAs) and other cytokine assays. At baseline, patients with schizophrenia and psychosis had higher levels of inflammatory cytokines, particularly IL-6 and TNF-α, compared to controls (Song et al., 2014; Noto et al., 2015). Treatment with anti-psychotics reduced these inflammatory cytokines (Song et al., 2014; Noto et al., 2015; Sobiś et al., 2015; Juncal-Ruiz et al., 2018) and, in some cases, increased the levels of anti-inflammatory cytokines, such as IL-10 (Sobiś et al., 2015). A meta-analysis of 23 studies investigating the effect of anti-psychotic treatment on peripheral cytokine levels in schizophrenia found reduced levels of IL-1β and IFN-γ, and increased plasma levels of soluble IL-2 receptor after treatment (Tourjman et al., 2013), similar to other studies shown in Table 3. Additionally, a meta-analysis of first-episode psychosis cohorts found that anti-psychotic treatment decreased peripheral levels of pro-inflammatory cytokines IL-1β, IL-6, IFN-γ, and TNF-α (Marcinowicz et al., 2021). The levels of anti-inflammatory IL-4 and IL-10 were also decreased following anti-psychotic treatment in this study, which contradicts some studies shown in Table 3.

**Table 3:** Anti-inflammatory effects of anti-psychotics in human studies and in studies using *in vitro* or *ex vivo* human cells in culture or stimulation assays.

**Drug name**	**Population**	**Method**	**Results: Difference in patients (vs. controls) at baseline, or patients pre- and post-treatment**	**References**
Risperidone	First episode schizophrenia (*n* = 62, 29 females, mean age 24.7 years) Healthy controls (*n* = 60, 27 females, mean age 26.2 years)	ELISA for serum IL-1β, IL-6, and TNF-α.	At baseline: ↑ IL-1β, IL-6, and TNF-α. On treatment: Transient ↓ IL-1β and IL-6 for 2 weeks but returned to pre-treatment levels by 6 months. Steady ↑ TNF-α.	Song et al., 2014
Risperidone	First episode psychosis (*n* = 55, 19 females, mean age 24.75 years) Healthy controls (*n* = 57, 27 females, mean age 26.6 years)	Cytokine flow cytometric bead array for serum. IL-2, IL-10, IL-4, IL-6, IFN-γ, TNF-α, and IL-17.	At baseline: ↑ IL-6, IL-10, and TNF-α. On treatment: ↓ and phosphorylation of IL-6, IL-10, and TNF-α.	Noto et al., 2015
Risperidone, aripiprazole	First episode psychosis (*n* = 75, 40) females, mean age 29.6) Healthy controls (*n* = 75, 36 females, mean age 28.7 years)	Magnetic bead panel for serum levels of 21 cytokines.	At baseline: ↑ IL-8 and MIP-1β. On treatment (similar for both drugs): **↓** IL-8, MIP-1β, Fractalkine, TNF-α, IL-7, IL-13, IL-17α, IL-23, and IL-21.	Juncal-Ruiz et al., 2018
Aripiprazole	Chronic schizophrenia (*n* = 17, seven females, mean age 51.1 years) No control group	ELISA for serum IL-1Ra, sTNF-R1, IFN-γ, IL-12 p40, IL-4, IL-1β, TNF-α, IL-23, insulin, IL-10, IL-6, and TGF-β1. Latex assay for serum CRP levels.	On treatment: ↓ CRP, insulin, IL-1β, IL-6, TNF-α, sTNF-R1, IL-12, IL-23, IL-1Ra, TGF-β1, IL-4, IFN-γ. ↑ IL-10.	Sobiś et al., 2015
Risperidone, olanzapine, aripiprazole	Schizophrenia (*n* = 27, 11 females, mean age 30.6 years) No control group	qPCR of PBMCs for *IL6, STAT3*, and *RORC* gene expression. Plasma multiplex assay for IL-1β, IL-6, IL-17A, IL-23, and IL-33.	ON TREATMENT: downregulation of *STAT3* gene expression. ↓ plasma IL-1β, IL-6, and IL-17A.	Subbanna et al., 2020
Clozapine	Schizophrenic inpatients (*n* = 10, all males, mean age not reported, longitudinal study, clozapine monotherapy) Schizophrenic patients (*n* = 15, sex and age data not reported, clozapine polytherapy, cross-section study) Control group (*n* = 25, sex and age data not reported, schizophrenic patients not on clozapine, cross section study)	Serum levels of high sensitive CRP (hsCRP) measured using high-sensitivity immunoturbidimetric assay	Following first-time use, hsCRP levels ↑ by 600% Cross-section analysis of long-term clozapine patients compared to controls did not show a statistical difference between groups, suggesting hsCRP elevation may be an acute response	Löffler et al., 2010
**Drug name**	**Population or cell type**	**Immunological or cellular method**	**Results** ***in vitro*****/*****ex vivo*****: Difference following application of drug**	**References**
Haloperidol, quetiapine, clozapine, risperidone	First episode schizophrenia (*n* = 12, six females, mean age 34 years) No control group	Patient PBMCs with LPS and poly(I:C) stimulation assays with drug incubation. ELISA for supernatant IL-4, IL-10, and IFN-γ.	After LPS stimulation: Haloperidol ↑ IL-4 and IL-10, and quetiapine ↑ IL-4. After poly(I:C)-stimulation: Clozapine and quetiapine ↑ IL-4 and IL-10, and risperidone ↑ IL-10. All anti-psychotics ↓ IFN-γ in LPS- and poly(I:C)-stimulation.	Al-Amin et al., 2013
Olanzapine, aripiprazole	Human PBMCs isolated from healthy blood donors (no demographic information available) Human monocytic cell line THP-1 cells	PBMCs and THP-1 cells drug incubation assay. qPCR for *IL1B, IL6, IL10, TGFB1, TNF*. ELISA for supernatant IL-1β, IL-6, IL-10, and TNF-α, and 27-plex cytokine/chemokine assay for PBMCs supernatant.	On treatment in PBMCs: **↓** mRNA IL-1β, IL-6, and TNF-α. Aripiprazole treatment only: ↑ IL-10 mRNA. **↓** supernatant IL-6, TNF-α, and IL-10 in ELISA. ↑ IL-2, IL-6, IFN-γ, IP-10, and MIP-1β in multiplex assay. On treatment in THP-1: **↓** mRNA and supernatant IL-1β and TNF-α.	Stapel et al., 2018
Clozapine, chlorpromazine, haloperidol, *N*-desmethylclozapine, and quetiapine	Whole blood culture of healthy controls (*n* = 10, all females, mean age 29.9 years)	Whole blood cultured with therapeutic concentration of each drug plus TSST-1 to stimulate cytokine production Flow cytometry to measure IL-1β, IL-2, IL-4, IL-6, IL-17, and TNF-α	↑ IL-17 following application with each drug ↑ TNF-α with chlorpromazine ↑ IL-2 with *N*-desmethylclozapine and quetiapine	Himmerich et al., 2011
Clozapine, haloperidol, chlorpromazine	PBMCs from healthy volunteers (*n* = 16, eight females, mean age 34.2 years)	Cultured and stimulated with LPS + PHA-M ELISA for IL-2, IFN-γ, IL-10, IL-4, IL-12, TNF-β, and TGF-β	↑ IL-10 and TGF-β with all drugs and ↓ IL-2 ↓ IL-4 and IFN-γ with chlorpromazine and haloperidol ↑ IFN-γ with clozapine	Szuster-Ciesielska et al., 2004

ELISA, enzyme-linked immunosorbent assay; IL= interleukin; TNF, tumor necrosis factor; MIP, macrophage inflammatory protein; IL-1Ra, IL-1 receptor antagonist; sTNF-R, soluble tumor necrosis factor receptors; IFN, interferon; CRP, C-reactive protein; PBMC, peripheral blood mononuclear cells; LPS, lipopolysaccharide; qPCR, quantitative polymerase chain reaction; *STAT*, signal transducer and activator of transcription; *RORC*, RAR-related orphan receptor C; mRNA, messenger ribonucleic acid; TSST, toxic shock syndrome toxin; TGF, transforming growth factor.

Several studies also used peripheral blood mononuclear cells (PBMCs) from patients with schizophrenia or healthy volunteers to conduct *in vitro* stimulation and signaling assays. Patient PBMCs showed decreases in IFN-γ and increases in IL-4 and IL-10 in cell culture supernatant after incubation with anti-psychotics (Al-Amin et al., 2013). One study also showed downregulation of *STAT3* gene expression, indicating that anti-psychotics reduced inflammatory cell signaling *via* the signal transducer and activator of transcription (STAT) pathways (Subbanna et al., 2020) (Figure 1). PBMCs from healthy volunteers showed decreases in IL-6 and TNF-α and increases in IL-10 when incubated with anti-psychotics *in vitro*, similar to the serum cytokine studies in patient cohorts (Stapel et al., 2018).

### 3.3. Mood stabilizers

Mood stabilizers, such as lithium, valproate, and lamotrigine, are commonly used to treat bipolar disorder and psychosis, with some evidence to support their use in ASD (Aman et al., 2008; Canitano, 2015). These drugs have complex mechanisms of action, all affecting neurotransmission in the brain (Rapoport et al., 2009; Chiu et al., 2013). The anti-inflammatory effects of commonly used mood stabilizers have primarily been investigated in adults with bipolar disorder, using ELISAs and other assays to measure peripheral cytokine/chemokine levels. Meta-analyses have shown higher levels of pro-inflammatory and anti-inflammatory cytokines, TNF-α, IL-1β, IL-6, IL-4, and IL-10 in patients with bipolar disorder compared to controls at baseline (Modabbernia et al., 2013). The studies reviewed in Table 4 found that lithium and lamotrigine decreased the levels of IL-6, IL-10, IFN-γ, and IL-1β, as well as C-reactive protein (CRP) (Boufidou et al., 2004; Shi et al., 2018; Queissner et al., 2021). Conversely, increased levels of TNF-α and IL-4 were found in euthymic bipolar patients on lithium monotherapy (Guloksuz et al., 2010). In general, mood stabilizing drugs have been shown to normalize elevated peripheral pro-inflammatory cytokine levels in bipolar disorder (van den Ameele et al., 2016). In cerebrospinal fluid (CSF), levels of 1L-8 were found to be increased in patients with euthymic bipolar disorder compared to controls, and this was positively associated with ongoing lithium and antipsychotic treatment (Isgren et al., 2015). *Ex vivo* studies (Table 5) using peripheral blood monocytes from patients with bipolar disorder have also shown that lithium reduces expression of inflammatory genes (*IL-6, TNF, CXCL2*) (Padmos et al., 2008) and decreases IL-6 and IL-1β production (Knijff et al., 2007). Similarly, in cultures from healthy volunteers, lithium reduced IL-6, IL-1β, IL-2, TNF-α, and IFN-γ, and increased IL-4, IL-10, and IL-22 (Rapaport and Manji, 2001; Himmerich et al., 2013). In human microglia cultures, lithium reduced IFN-γ and STAT1/STAT3 signaling (Göttert et al., 2022). Valproic acid had similar effects on cytokines as lithium, and also reduced differentiation of T helper (Th) 17 cells and dendritic cells, chemotaxis migration of dendritic cells, lymphocyte proliferation, NF-κB activation, and nuclear levels of IFN regulatory factors (Nencioni et al., 2007; Leu et al., 2017) (Figure 1).

**Table 4:** Anti-inflammatory effects of mood stabilizers in human studies.

**Drug name**	**Population**	**Method**	**Results: Difference in patients vs. controls at baseline, or patients pre- and post-treatment**	**References**
Lithium	Bipolar affective disorder on chronic lithium treatment (*n* = 40, 20 females, mean age 42.8 years) Bipolar affective disorder drug naïve (*n* = 10^*^) Healthy controls (*n* = 20, 10 females, mean age 49 years)	ELISPOT for IL-2, IL-6, IFN-γ, and IL-10 in PBLs. ELISA for serum IL-2, IL-6, IL-10, IFN-γ.	On treatment: ↓ IL-2, IL-6, IL-10 and IFN-γ secreting cells in chronic lithium patients. ↓ number of cytokine secreting cells in drug naïve patients after 3 months on lithium. *In vitro* stimulation of lymphocytes with lithium did not affect the number of cytokine secreting cells.	Boufidou et al., 2004
Lithium	Euthymic bipolar disorder, medication free (*n* = 16, four females, mean age 32.3 years) Euthymic bipolar disorder, lithium monotherapy (*n* = 15, four females, mean age 31.8 years) Healthy controls (*n* = 16, four females, mean age 31.8 years)	Cytometric bead array kit for serum IFN-γ, TNF-α, IL-2, IL-4, IL-5, and IL-10.	On treatment: No differences in cytokine levels. TNF-α and IL-4 levels in lithium monotherapy euthymic bipolar patients were higher than in both the medication free euthymic bipolar patients and controls.	Guloksuz et al., 2010
Lithium	Bipolar disorder (*n* = 267, 128 females, mean age 44 years) No control group	CRP latex assay and chemiluminescence immunoassay.	On treatment: negative correlation between duration of lithium treatment and CRP levels (i.e., reduction in CRP with longer duration of lithium). ↑ CRP in males with symptom progression compared to males without.	Queissner et al., 2021
Lamotrigine, valproate	Depression or recurrent bipolar disorder divided into either lamotrigine or valproate group (*n* = 70 in each group, 36 females in each group, mean age 48.4 years) Healthy controls (*n* = 70, 35 females, mean age 48.7 years)	ELISA for serum macrophage MIF, IL-1β and IL-6.	At baseline: No difference between groups On treatment: ↓ macrophage MIF, IL-1β, and IL-6 for both drugs compared to baseline. ↑ in all cytokines in lamotrigine group compared to valproate.	Shi et al., 2018
Lithium and antipsychotics	Euthymic bipolar disorder (*n* = 121, 74 females, mean age 36 years) Healthy controls (*n* = 71, 45 females, mean age 32 years)	Singleplex assay for CSF IL-6. Multi-array and multi-spot human cytokine assay for CSF IL-1β, IL-2, IL-4, IL-5, IL-8/CXCL8, IL-10, IL-12, IL-13, TNF-α, and IFN-γ.	On treatment: ↑ IL-8, associated with ongoing lithium and antipsychotic treatment	Isgren et al., 2015

* No other demographic information available.

ELISPOT, enzyme-linked immunospot assay; PBLs, peripheral blood lymphocytes; IL, interleukin; IFN, interferon; TNF, tumor necrosis factor; CRP, C-reactive protein; ELISA, enzyme-linked immunosorbent assay; MIF, migration inhibitory factor; CSF, cerebrospinal fluid.

**Table 5:** Anti-inflammatory effects of mood stabilizers using *in vitro* or *ex vivo* human cells in culture or stimulation assays.

**Drug name**	**Population or cell type**	**Method**	**Results: Difference in patients vs. controls at baseline, or patients pre- and post-treatment** **Results *in vitro*/*ex vivo*: Difference following application of drug**	**References**
Lithium	PBMCs from bipolar patients (*n* = 42, 26 females, mean age 42 years) and healthy controls (*n* = 25, 14 females, mean age 40 years)	qPCR of PBMCs using *Taq*Man probes for inflammatory (*PDE4B, IL1B, IL6, TNF, TNFAIP3, PTGS2*, and *PTX3*), trafficking (*CCL2, CCL7, CCL20, CXCL2, CCR2*, and *CDC42*), survival (*BCL2A1* and *EMP1*), and MAPK pathway (*MAPK6, DUSP2, NAB2*, and *ATF3*) genes.	Baseline: ↑ inflammatory genes *PDE4B, IL1B, IL6, TNF, TNFAIP3, PTGS2, PTX3, CCL2, CCL7, CCL20, CXCL2, CCR2, CDC42, MAPK6, DUSP2, NAB2*, and *ATF3*. On treatment: ↓*PDE4B, TNF, IL1B, IL6, TNFAIP3, PTGS2, PTX3, CCL20, CXCL2, BCL2A1*, and *DUSP2*. Levels of mRNA reduced but remain above those of healthy controls.	Padmos et al., 2008
Lithium	Peripheral blood monocytes from bipolar disorder patients (*n* = 80, 40 females, mean age 44 years; *n* = 64 lithium-treated) Healthy controls (*n* = 59, 33 females, mean age 38 years)	ELISA for supernatant IL-1β and IL-6.	Monocytes from non-lithium-treated bipolar patients: LPS-stimulation induced abnormal IL-1β/IL-6 production ratio (low IL-1β and high IL-6)- this ratio was restored with lithium treatment. *In vitro* exposure to lithium: ↑ IL-1β, but no change to IL-6 production.	Knijff et al., 2007
Lithium	Whole blood cultures from healthy volunteers (*n* = 10, four females, mean age 36.9 years)	Whole blood culture and 5-day incubation with lithium. Commercial assays for supernatant IL-6, IL-2, IL-4, IL-10, IFN-γ, and cytokine receptors.	Lithium incubation ↑ IL-4 and IL-10, ↓ IL-6, IL-2, and IFN-γ.	Rapaport and Manji, 2001
Carbamazepine, lamotrigine, valproic acid, lithium	Whole blood cultures from healthy female volunteers (*n* = 14, mean age 29 years)	TSST-1 stimulation assay with drug incubation. Bead array flow cytometry for supernatant IL-1β, IL-2, IL-4, IL-6, IL-17, and TNF-α. ELISA for supernatant IL-22.	All drugs ↓ TNF-α. ↓ IL-1β following incubation with carbamazepine, lamotrigine, and lithium. ↓ IL-2 following incubation with carbamazepine, lamotrigine, and valproic acid. ↑ IL-22 following incubation with, carbamazepine and lithium, but ↓ with valproic acid.	Himmerich et al., 2013
Lithium	Immortalized human microglia cell line (HM-IM) Primary human microglia from brain biopsies of epileptic patients (*n* = 14, five females, mean age 33.2 years) Microglia-like cells from hIPSCs	Immunofluorescence, qPCR, Western blots. Cytokines measured with Multi-Spot Assay System. Chromatin immunoprecipitation. Liquid chromatography for tryptophan and kynurenine concentrations	Lithium ↓ IFN-γ-induced IDO-1 mRNA and protein expression, and ↑ STAT1/STAT3 expression and binding to IDO1. Lithium ↓ LPS-induced IDO-1 activity in primary microglia, while ↑ IL-10 production.	Göttert et al., 2022
Valproate, carbamazepine	THP-1 cells	Electrophoretic mobility shift assay. ELISA for TNF-α, and IL-6.	Valproate ↓ LPS-induced NF-κβ activation in dose-dependent manner, and ↓ LPS-induced TNF-α and IL-6 (other drugs no differences found).	Ichiyama et al., 2000
Valproate	Human monocyte-derived DCs from healthy volunteers^*^	Flow cytometry. Mixed leukocyte reaction. ELISA for IL-6, IL-10, IL-12, and TNF-α. Transwell migration assay Immunoblotting.	Valproate skews DC differentiation by preventing expression of DC marker CD1a and affecting the expression of co-stimulation and adhesion molecules. ↑ capacity to stimulate allogeneic lymphocyte proliferation. ↑ chemotaxis DC migration toward CCL19 in Transwell assay. ↑ RelB protein (NF-κB subunit) in nuclear extracts, blocked poly(I-C)-induced RelB nuclear re-localization in a dose-dependent manner. ↑ nuclear levels of interferon regulatory factors IRF3 and IRF-8 in poly(I-C)-stimulated DCs	Nencioni et al., 2007
Valproate, lithium	Monocyte-derived DCs from healthy volunteers^*^	LPS stimulation assay. ELISA for supernatant IL-6, IL-10, TNF-α, IL-8, IL-17, and IL-23p19/p40.	Both drugs ↑ IL-6 and TNF-α, but only lithium ↑ IL-10. Valproate ↑ IL-8 but ↑ IL-10 and IL-23. Valproate ↓ capacity of LPS-stimulated DCs to promote the differentiation of Th17 cells.	Leu et al., 2017

* No other demographic data available.

PBMCs, peripheral blood mononuclear cells; qPCR, quantitative polymerase chain reaction; MAPK, mitogen-activated protein kinase; mRNA, messenger ribonucleic acid; ELISA, enzyme-linked immunosorbent assay; LPS, lipopolysaccharide; IL, interleukin; TSST-1, toxic shock syndrome toxin; TNF, tumor necrosis factor; hIPSCs, human induced pluripotent stem cells; STAT, signal transducer and activator of transcription; IDO, indoleamine 2; 3-dioxygenase, DCs, dendritic cells.

**Table 6:** Anti-inflammatory effects of other drugs in human studies and in studies using *in vitro* or *ex vivo* human cells in culture or stimulation assays.

**Drug name**	**Population or cell type**	**Method**	**Results: Difference in patients vs. controls at baseline, or patients pre- and post-treatment Results *in vitro*/*ex vivo*: Difference following application of drug**	**References**
Methylphenidate, dexamphetamine, or atomoxetine monotherapy	ADHD patients, children and adults: children (*n* = 49, 13 females, mean age 13 years), adults (*n* = 105, 74 females, mean age 36 years) Healthy controls: children (*n* = 4, one female, mean age 13 years), adults (*n* = 57, 36 females, mean age 38 years)	Sandwich immunoassay for plasma CRP, serum amyloid A, sICAM-1, and sVCAM-1.	At baseline: ↑ sICAM-1 and sVCAM-1 in all patients vs. controls (both children and adults). On treatment: Children with ADHD on medication ↓ sICAM-1 and sVCAM-1, compared to control groups of both children and adults.	Yang et al., 2020
Methylphenidate	Children with ADHD (*n* = 130, 28 females, mean age 9 years) Healthy controls (*n* = 49, 16 females, mean age 10 years, included siblings of patients)	Liquid-chromatography–tandem mass spectrometry (LC–MS/MS) to measure levels of tryptophan, kynurenic acid, anthranilic acid, xanthurenic acid, quinolinic acid, and nicotinamide.	Baseline: Similar levels of kynurenic acid and xanthurenic acid between ADHD and controls. On treatment: ↑ plasma kynurenic acid and xanthurenic acid.	Molina-Carballo et al., 2021
Clonidine	Whole blood cultures from preterm infants (*n* = 7, three females, ≤ 30 weeks gestational age) and full-term (*n* = 19, six females, ≥37 weeks gestational age)	ELISA for supernatant IL-1β, IL-6, IL-8, IL-10, IL-12p70, and TNF-α.	Clonidine at < 10^−9^ M ↑ IL-6, while at >10^−5^ M ↑ IL-1β and ↓ TNF-α levels.	Chavez-Valdez et al., 2013
Ketamine	PBMCs from healthy male volunteers (*n* = 10, mean age unavailable)	PMA and ionomycin stimulation of PBMCs with ketamine. T cell subsets measured by flow cytometry. ELISA for supernatant IFN-γ and IL-4. Electrophoretic mobility shift assay for T-bet and GATA3 DNA binding activity.	Ketamine ↑ proportion of Th1 and Th2 subsets but increased Th1/Th2 ratio in the presence of PMA and ionomycin. Ketamine ↓ IFN-γ and IL-4, and ↑ T-bet and GATA3 but ↑ T-bet/GATA3 ratio.	Gao et al., 2011
Ketamine	HUVECs with high glucose incubation (model of endothelial inflammation)	Ketamine incubation in HUVEC culture. Adhesion assays. ROS assays. Western blotting.	Ketamine ↓ high-glucose-induced monocyte/endothelial adhesion and adhesion molecule expression, in a dose-dependent manner. Ketamine ↓ high-glucose-induced NF-κB and IκBα activation, phospho-PKC Ser660 phosphorylation, and ROS production.	Wang et al., 2020

ELISA, enzyme-linked immunosorbent assay; IL, interleukin; TNF, tumor necrosis factor; PBMCs, peripheral blood mononuclear cells; PMA, phorbol 12-myristate 13-acetate; Th, T helper cells; HUVECs, human umbilical vein endothelial cells; ROS, reactive oxygen species; ADHD, attention-deficit/hyperactivity disorder; CRP, C-reactive protein; sICAM-1, soluble intercellular adhesion molecule 1; sVCAM-1, soluble vascular cell adhesion molecule 1.

### 3.4. Other psychiatric drugs

There is a smaller literature regarding the anti-inflammatory effects of other psychiatric drugs such as stimulants, α-agonists, and glutamatergic drugs. Studies investigating the anti-inflammatory effects of common ADHD drugs (e.g., methylphenidate, dexamphetamine, or atomoxetine) in humans are limited. One study found that these drugs increased levels of soluble ICAM-1, and soluble VCAM-1 (sICAM-1 and sVCAM-1, respectively) in children with ADHD (Yang et al., 2020), contrasting with the published effects of SSRIs on adhesion molecules. Methylphenidate has also been shown to increase plasma KYNA and xanthurenic acid in children with ADHD (Molina-Carballo et al., 2021). As previously mentioned, KYNA is anti-inflammatory, and this increase is similar to the effect of SSRIs shown by Halaris et al. (2015). Clonidine is an α2 adrenergic agonist and primarily used as an anti-hypertensive agent; however, it is also effective in treating ADHD and behavioral disturbances in neurodevelopmental disorders (Ming et al., 2008; Kollins et al., 2011). Studies investigating the biological effects of clonidine in humans in the context of ADHD are limited. In an *in vitro* study of whole blood cultured from infants, incubation with clonidine increased the levels of pro-inflammatory cytokine IL-1β but reduced TNF-α, similar to the effects of lithium.

Ketamine is a non-competitive antagonist at the N-methyl-D-aspartate (NMDA) receptor and commonly used as an analgesic in pain management (Visser and Schug, 2006). Ketamine has been shown to have dopaminergic and anti-depressant effects and is sometimes used to treat complex neuropsychiatric symptoms, with an emerging role in depression (Kokkinou et al., 2018). Human studies examining the anti-inflammatory effects of ketamine are very limited. In PBMCs from healthy males, ketamine decreased the proportion of Th1 and Th2 cell subsets but increased the Th1/Th2 ratio (Gao et al., 2011). Ketamine also decreased expression of transcription factors T-bet and GATA3, thereby preventing Th1 and Th2 cell differentiation. Subsequent cytokine production was also reduced, demonstrated by decreased levels of IFN-γ (Th1-produced cytokine) and IL-4 (Th2-produced cytokine) (Gao et al., 2011). In Human Umbilical Vein Endothelial Cells (HUVECs), ketamine reduced high-glucose-induced monocyte/endothelial adhesion, while also reducing NF-κB activation and reactive oxygen species (ROS) production (Wang et al., 2020).

## 4. Discussion

Our review highlights that commonly used psychiatric drugs, namely anti-depressants, anti-psychotics, and mood stabilizers, have demonstrated anti-inflammatory effects in humans. In general, there is evidence to suggest that all drug classes reduce peripheral levels of pro-inflammatory cytokines (particularly, IL-6 and TNF-α) and decrease inflammatory pathway signaling (e.g., NF-κB, STAT). However, the literature to date is limited in scope, both clinically and biologically. Most studies have been conducted in adult cohorts, mostly in depression, psychosis, and bipolar disorder. Although these drugs are commonly used in children to treat neurodevelopmental and neuropsychiatric symptoms, there is a lack of pediatric studies in this context. Disorders with relatively strong support for the role of inflammation, such as ASD (Meltzer and Van de Water, 2017) and post-traumatic stress disorder (Passos et al., 2015), have had limited investigation into the anti-inflammatory effects of psychiatric drugs. Though they are used very commonly, there is particularly limited human evidence regarding ADHD drugs (e.g., methylphenidate, dexamphetamine, or atomoxetine); however, they have shown anti-inflammatory effects in animal studies (Aga-Mizrachi et al., 2014; Yssel et al., 2018).

In addition, the methodology used to demonstrate anti-inflammatory effects is restricted: most studies only examine the levels of cytokines in peripheral blood before and after treatment, and *in vitro* and *ex vivo* studies likewise tend to examine cytokines or cytokine signaling. While cytokine and chemokine measures are commonly used as clinical indicators of inflammation, it is important to note that the stability of these measures can be affected by many factors, including sample collection issues, duration and method of storage, time of collection, fasting, physical activity and stress. Cytokine levels can vary substantially between serum, plasma, whole blood, and CSF. Additionally, the method of analysis, for example ELISAs compared to multiplex assays, can also influence the quantification results (Leng et al., 2008; Zhou et al., 2010; Liu et al., 2021). As there are no standardized, gold-standard guidelines on how to reliably quantify cytokines, results regarding cytokine data should be interpreted and compared with caution. Studies of the CNS would also be of immense value as this field moves forward. Studies have begun to investigate markers of inflammation in CSF in mental health disorders, yet studies measuring CSF following use of a therapeutic drug are limited. A study by Miller et al. (2017) showed that despite pregnant women taking SSRIs having significantly reduced levels of IFN-γ and IL-8 in peripheral blood compared to pregnant women not using SSRIs, no significant differences were observed in CSF. A meta-analysis of markers of central inflammation in MDD patients determined CSF levels of IL-6 and TNF-α were higher in MDD patients compared to controls, and there was no significant correlation between abnormalities in CSF and those seen in peripheral blood (Enache et al., 2019), further complicating understanding the mechanisms underlying these conditions and how pharmacological agents may interact. The literature on inflammatory metabolism (e.g., kynurenine pathway), and epigenetic regulation of immune cells, is very limited, but provides opportunities for future studies.

Another remaining key issue is whether inflammation is relevant to all patients with mental health disorders, or only in specific subgroups. Resolving this is paramount to allow targeted therapeutics to be developed and implemented clinically in the future. Studies targeting pro-inflammatory cytokines, such as TNF-α, have exemplified this problem (Raison et al., 2013). In a study of refractory depression, infliximab, a TNF-α inhibitor, did not have generalizable benefit in the treatment of refractory depression, but did result in clinical benefits in patients with elevated CRP at baseline. This study supports the “immune subgroup” hypothesis in mental health disorders, emphasizing the need for patient subgrouping and individualized therapies. As well, as mentioned previously, the neurotransmitter hypotheses relating to many mental health disorders are not conclusive. While there is significant evidence supporting neurotransmitter dysfunction in these contexts, the origins of these dysfunctions are ambiguous, and it is unclear whether neurotransmitter imbalance in mental health disorders is primary or secondary to other processes.

A final question, and one not addressed by the current literature, is the issue of whether inflammation has a causal pathway in the generation or perpetuation of mental health disorders, as opposed to an “association” only. None of the human studies we have reviewed here showed causation between inflammatory states and mental health symptoms, only association. While animal models have convincingly shown (neuro)inflammation causes and exacerbates mental health disorders, and ameliorating inflammation produces symptomatic benefits, this degree of evidence is largely absent in humans, to date (Hayley, 2011; Song and Wang, 2011; Harvey and Boksa, 2012; Gumusoglu and Stevens, 2019). Hence, even though there is evidence that commonly used psychiatric drugs have anti-inflammatory properties, there is no evidence in human patients of clinical benefit being a direct result of reduced inflammation. Similarly, there are minimal comparative studies between anti-inflammatory properties of drugs between classes (e.g., comparing SSRIs/SNRIs with anti-psychotics), and no studies exploring augmentative properties (i.e., additive anti-inflammatory effects) of multiple psychiatric drug classes in patients.

In summary, we have presented emerging evidence for the anti-inflammatory properties of commonly used psychiatric drugs. As researchers, clinicians, and patients and their families become increasingly aware of the role of inflammation in brain health, there should be a degree of comfort in the idea that drugs used to treat common mental health symptoms may also abrogate this inflammation.

## Author contributions

SP and BK: conceptual design, literature review, and primary writing and editing of manuscript. RD: conceptual design and primary writing and editing of manuscript. All authors contributed to the article and approved the submitted version.
